# A qualitative exploration of substance misuse among homeless women in Addis Ababa, Ethiopia

**DOI:** 10.1186/s12888-020-02626-9

**Published:** 2020-05-06

**Authors:** Kibrom Haile, Halima Umer, Getinet Ayano, Edao Fejo, Tolesa Fanta

**Affiliations:** 1 Research and Training Department, St Amanuel Mental Specialized Hospital, Addis Ababa, Ethiopia; 2 Clinical Department, St Amanuel Mental Specialized Hospital, Addis Ababa, Ethiopia

**Keywords:** Substance use, Homeless, Women, Determinants, Sleeping rough

## Abstract

**Background:**

Substance use among homeless people is higher compared to the general population. In some studies, reported rates of problematic drug use among the homeless vary, with estimates ranging from 25 to 70%. There is a common perception that substance abuse and homelessness are linked, but there is considerable debate about the direction of the relationship. Despite observations of high levels of substance use among the homeless population in Addis Ababa, there are limited studies to date conducted on the topic. This study aims to explore the factors associated with onset of substance use and its continued use, patterns of substance use and its social and health consequences among female residents of a shelter in Addis Ababa, Ethiopia.

**Methods:**

A qualitative study was conducted in 2019. In-depth interviews were conducted on 14 study participants who were selected purposively. The qualitative data analysis software QDA Miner 5.0.30 was used for data processing and analysis.

**Results:**

Four major thematic areas were identified and they comprised the categories under which sub-themes were identified and coded. The major segments or categories included the following: reasons for the onset of substance use after becoming homeless, experiences of substance use and reasons for continued use, the harms which resulted on them from substance use, and the means of obtaining supply of the substances.

**Conclusion:**

Factors related to life on streets were strong reasons for onset of substance use, as well as for its continued use. Homeless women suffered untimely death, addiction, and ill health from use of substances; however, they gave priority to obtaining substances than any other thing, and used every means to grab a supply of the substances.

## Background

### Substances most commonly misused in Ethiopia

In Ethiopia the commonly used substances are alcohol, cigarette, khat and cannabis which frequently lead to addiction [1]. Khat (*Catha edulis*) is an evergreen shrub that is planted & cultivated in eastern through southern parts of Africa and the Arabian Peninsula [2]. The biochemically active constituents of khat responsible for its psycho-stimulant activity are the alkaloid chemicals called cathinone and cathine (a less potent form of cathinone) which are similar to the psychoactive amphetamine both structurally and functionally [3]. Fresh leaves of the shrub contain both of the alkaloids, and they are chewed by users to get the psycho-stimulant effect [4]. Khat is a legal substance like cigarette and alcohol in Ethiopia and it is openly sold at markets and chewed in streets [5]. In Ethiopia, chewing of khat is becoming habitual and increasing at an alarming rate. According to the 2016 Ethiopian Demographic and Health Survey report, 12% of women and 27% of men reported having ever chewed khat [6].

In Ethiopia, cannabis (Cannabis sativus) is an illegal substance and, in Addis Ababa, the police repeatedly apprehend dealers and abusers [7]; however, it is being cultivated in central, western and eastern parts of Ethiopia, and cannabis smoking is escalating in the urban areas [7]. The substances most frequently used by the homeless people in Ethiopia based on studies conducted on street youth are alcohol, cigarettes, khat, cannabis, snif benzene, snif glue, snif paint [7]. The use of hard illicit drugs such as cocaine and heroin is limited to drug traffickers, commercial sex workers and those with the financial means and access [8]; their use in Ethiopia, and among the homeless people is generally negligible.

### The burden and type of substance misuse among the homeless, including women

Substance use among homeless people is higher compared to the general population; in fact, it has been found to be considerably high according to several studies [9–15]. Substance use among the young homeless has been reported to be two to three times higher than that of the general population. Among the youth population of the homeless, marijuana has been identified as the drug of choice; and among street homeless female adolescents the rate of alcohol dependence was 31.3, and 44.0% ever injected drugs [16, 17]. In a study conducted in Canada, 82.4% of adult homeless women had at least one type of current substance use disorder; 70.5% of them had drug dependence and 37.8% had alcohol dependence [18]. According to studies, reported rates of problematic drug use among the homeless vary, with estimates ranging from 25 to 70% [18–21]. Variations are due probably to variations in the definitions of ‘problematic use’, and of ‘homelessness.’ In a meta-analysis of 29 studies conducted from 1979 to 2005, the pooled prevalence of alcohol and drug dependence was 37.9 and 24.4%, respectively [22, 23]. A study conducted in the United States found out that homeless adult women have been constantly identified to have higher rates of alcohol and drug use than other women. The substances most commonly used by homeless women were alcohol, marijuana, and cocaine [21]. The study identified that 27.6% of homeless women had engaged in binge drinking, and 51.2% had used drugs [21].

Studies which were conducted on the street homeless in Addis Ababa identified almost all (93.2%) had ever used substances, with 10% having substance use disorders [24]. The prevalence of problematic alcohol use among the homeless people in Addis Ababa was found to be as high as 60% in one study [22]. Studies show that the likelihood of using more than one substance is high among the homeless. In one study, the majority (up to 72%) of current drug users reported using more than one drug [25]. Drug use patterns and trends vary across populations with different areas showing higher prevalence for some substances than others; and the trend shows change over time [26, 27]. Studies reporting substance misuse among homeless women in Ethiopia are scarce.

### How is substance misuse related to homelessness?

There is a common perception that substance abuse and homelessness are linked, but there is considerable debate about the direction of the relationship [28–31]. Some studies indicate that substance abuse is a risk factor for homelessness, whereas others suggest that homelessness induces drug use [30]. Personal drug use (19%) was cited as the second most common reason for becoming homeless; in fact, the majority of those who became homeless first used drugs before becoming homeless (87%) [25]. Johnson and Chambelain (2008) found out in their study that 43% of their study participants who were homeless had substance abuse problems, and 15% of them had had substance abuse problems prior to becoming homeless for the first time. Johnson and Chamberlain (2008) also indicated that two thirds (66%) developed problematic substance use after they became homeless [32]. Substance use may increase the risk of homelessness by undermining people’s social ties and economic stability [33]. Earlier age (before age 25 years) at homelessness onset, and the duration of life time as homeless were related to increased drug use among homeless adults [11, 34, 35]. Regarding the factors associated with substance use among homeless people, a study identified that living in the streets (no shelter), high stress scores, high adverse childhood experiences (ACE) scores, race, and gender were significantly associated with current use of substances [36].

Drug using homeless populations suffer a lack of social connectedness and their personal safety is at risk. There is a higher rate of drug-related deaths among homeless populations compared with the general population [26]. Substance use has been identified as the main mental health problem for homeless people [23]. Drug use is believed to be an important factor contributing to the poor health and increased mortality risk that has been widely observed among homeless individuals [37, 38]. Drug using homeless people suffer from numerous adverse health effects, including overdoses, psychiatric conditions, and infectious diseases [39, 40]. Substance use among homeless populations has consistently been associated with a number of adverse outcomes, such as premature mortality [41], symptoms of mental illness [11, 42], and longer duration of homelessness [43–48]. Mental ill health is strongly associated with homelessness as both a cause and a consequence [26]. The mortality rate among homeless youth is 10 times higher than that in housed youth, with drug overdose being one of the leading causes of death [49].

### The need to conduct this study

Despite observations of high levels of substance use among the homeless population in Addis Ababa, Ethiopia, there are limited studies conducted on the topic till the date. Particularly, in the context of homeless women, the database can be considered non-existent. Even though the prevalence of substance use among homeless females is lower than that of homeless males [7], the recognition of the problem among female homeless people will result in an in-depth understanding of the problem. The current study aims to explore the factors associated with onset of substance use and its continued use, patterns of substance use and its social and health consequences among female residents of a shelter in Addis Ababa, Ethiopia. The purpose of the study is to generate data to enable general understanding of the characteristics of substance use among homeless women, and to also lay the foundation upon which further research can be conducted. For the purpose of our study ‘homelessness’ indicates only those sleeping in designated public spaces or shelters, or those who are ‘roofless.’

## Methods

A qualitative study was conducted in 2019 to explore the determinants of substance use among female residents of a shelter in Addis Ababa. The shelter is one of the eight similar shelters built in Addis Ababa over the past few months. The shelter is funded by the Addis Ababa Administration’s Bureau of Labor and Social Affairs. It is run jointly by the Bureau and volunteer individuals. The shelter provided services for up to 250 people at the time of the study; the beneficiaries of the shelter services were adult females and elderly men. The shelter provided food, clothing and shelter, as well as medical, mental health and psychosocial services. This study was part of a bigger study conducted by the first author to explore pathways through homelessness among women. A purposive sampling technique of typical sampling was conducted in which respondents who had rich experience of life as homeless were included for interview. In-depth interviewing was employed to collect data from participants.

### Study setting

The study was conducted in Addis Ababa, which is the capital and largest city in Ethiopia. The city is located close to the center of the country on a plateau at an altitude of 2408 m above sea level. The city is administratively divided into 10 sub-cities. The current population size is estimated at 5–7 million. The city is inhabited by numerous nations and nationalities of Ethiopians, with only 1% of the people being foreigners. Females account for 54.2% of the population of Addis Ababa [50]. Yearly, significant number of people migrate from the smaller cities and the rural areas of Ethiopia to Addis Ababa. The population of Addis Ababa is a mixture of all the ethnic groups in the country. As the City is the major economic and political center of the country, people tend to migrate to it in search for better opportunities. In Addis Ababa, the city administration estimates the number of homeless individuals to be around 50,000 [24].

### Sampling and data collection procedure

One-on-one in-depth interviews were conducted to collect data from participants by using open-ended interview guide questions. Purposive sampling with typical sampling technique was employed to include participants who had rich experience as homeless. Participants who could narrate their experiences well were selected based on the historical accounts they gave at the time of admission into the shelter. Those with active symptoms of mental illness were excluded from the study. Total sample was determined at the point theoretical saturation was reached. A total of 15 women were interviewed, but one of the interview data was not included in the analysis due to data quality issues. All interviews were audio-recorded by using a digital voice recorder on a smart phone.

### Data analysis

The data was analyzed using the qualitative data analysis software QDA Miner 5.0.30. The recorded audio was first transcribed into text written in the local language Amharic. The text was read line-by-line and then translated into English language, with data for each respondent made into word document which was imported into the software as ‘cases’. A careful line by line reading of each document was done to identify major themes within the data; the themes were then labeled and entered into the ‘variables’ section of the software. The sub-thematic concepts emerging from the content of the responses were coded and included under each relevant segment or ‘variable’ for further analysis.

## Results

Four major thematic areas were identified and they comprised the major segments or ‘categories.’ The categories included the following themes: the substances used and the reason for the onset of their use, the experiences of substance use and the reasons for continued use, the harms of substance use, and the means of obtaining a supply of the substances. During data analysis, emerging themes were coded and categorized under each ‘variable.’ All the participants began substance use, at least with the current pattern for alcohol only, after they became homeless. The substances used by the homeless women were khat, cigarettes, cannabis, alcohol and inhalants (snif glue & snif benzene). The major thematic categories and the codes under them are discussed.

### The substance used and the reasons for the onset of their use

Pressure from peers was an important reason for initiating substance use among the respondents. Their homeless associates were the main reason for the onset of substance use. Next are the responses by the participants:

*“Khat is the first psychoactive substance I started using. It was after I started living on the streets. I started by pressure from a friend. My girlfriend, who uses khat, encouraged me to chew khat the first time by telling me to chew the khat together with caramel so that it wouldn’t be bitter for me; later on I was used to chewing khat like others. Then I started smoking cigarette after bouts of choking and coughing at the beginning till I got used to it.” [1*^*st*^*participant]*

*“ … … … . we found children on the street who wore old ragged clothes and were holding “amag” (‘amag’, also called snif glue, is an inhalant psychoactive substance which is found in adhesive fluid which is used to fix shoes) and using it. She (my friend) knew the children. I asked her what they were holding and using and she told me it was “amag” and told me I had to taste it. I didn’t know what it was but curiously thought it was juice; I took a lot of it in my mouth. She later on told me about the substance and how it can be used; finally, after sometime, I got used to it. Next to “amag” I started smoking cigarette. Then followed khat, cannabis, and alcoholic drinks. Thereafter, I continued using those substances almost daily.” [5*^*th*^*participant]*

*“The first day they took me to their place, I saw them chew khat, smoke cigarettes, and drinking alcoholic beverages. On the third day, they persuaded me to drink alcohol. I resisted at first, but finally one of my female friends almost coerced me to drink alcohol. The alcohol they gave me was a mixture of different strong alcoholic spirits; it was my first time to drink that kind of alcohol with that amount. The psychoactive substances I later continued to use were cigarettes, khat, and alcoholic drinks.” [9*^*th*^*participant]*

Distressing events resulted in onset of substance use in the study participants. The distressing events were considered the reason for the onset of substance use. The respondents described it as follows:

*“The day I realized I was exploited by the last boyfriend I had, I was so upset I unusually started drinking ‘tej’ (‘tej’ is a traditionally brewed honey wine.) I use tobacco, khat, alcoholic drinks, and sometimes cannabis.” [1*^*st*^*participant]*

*“I use all kinds of psychoactive substances. The substances I use are cigarettes, ‘amag’, khat, cannabis and alcoholic drinks. I started using all substances after I was on the street. The reason I started using substances was to cope with the stress created by the challenges of life on the street.” [15*^*th*^*participant]*

Conforming to street culture was a motive for the participants to begin using substances. The respondents described:

*“I did what my associates of the street did: I smoked cigarettes and chewed khat. I started using psychoactive substances to look alike my street friends; I felt I should do whatever they do and that includes using substances. The substances that I use are cigarettes, khat, cannabis (ganja), ‘amag’, and sometimes alcoholic drinks.” [8*^*th*^*participant]*

*“Once someone is living on the streets, being addicted to one or more of the substances is inevitable for him/her. This is because, since the street guys are using it, it is difficult for anyone to say no to using it.” [5*^*th*^*participant]*

Another reason for onset of substance use was that some respondents were induced to it since childhood; a participant who was born into the street responded as follows:

*“Since I was born and raised on the street, I have used psychoactive substances ever-since I can remember. I used snif benzene, ‘amag’, cigarette smoking, khat, the local alcoholic beverage ‘tej’ (local honey wine which we also call ‘ache’ by code of the street), and cannabis. I began using them all when I was as little as not to be able to remember when I first used them.” [6*^*th*^*participant]*

### Experiences of substance use and the reasons for continued use

The other major thematic area which emerged from the study was about the experiences of the respondents with substance use, and their reasons for continued use of the substances. The codes under this category are discussed.

Addiction was an experience, as well as reason continued use identified during the data analysis. Respondents gave the following accounts:

*“Early in the morning I have to smoke cigarette, otherwise I wouldn’t be able to open my eyes. I save a cigarette for early morning (5:00 AM) when I smoke it to help open my eyes.” [1*^*st*^*participant]*

*“I smoke first thing in the morning, I will be unable to even open my eyes; I wouldn’t be able to see anything otherwise.” [6th participant].*

*“On the streets, substances are more valuable than even your child; you chose to quench your addiction, and neglect your crying baby. Using the substance at need is like eating your food after a lengthy hunger.” [8th participant].*

Participants also described need to cope with stress as their experience and motive to use substances on an ongoing basis.

*“The only psychoactive substance I use is alcohol. I drink especially tej (local honey wine). I drink because I have stress and frustrations which I want to alleviate. I drink to forget myself (forget the miseries of life); the alcohol makes me relax.” [3*^*rd*^*participant]*

*“Cannabis (‘baye’ as we call it by code of the street) relives my stress and gets me relaxed; it also calms me when I am angry.” [6*^*th*^*participant]*

*“The benefits I get from using those substances while I lived on the streets are: I see lots of disgusting things on the streets and substances help me forget those events … ..I usually felt hopeless as if I am unwanted person; I also frequently felt hatred toward life because I felt like I had no identity, that was like feeling empty. Using substances relieved me of that kind of feeling; Smoking cigarettes relieves my stress.” [9*^*th*^*participant]*

*“ … … … ... The benefits I get by using substances on streets: khat relieves me of anger and frustration. I feel anger and frustration at my fate and the life I was forced into.” [12*^*th*^*participant]*

Respondents mentioned that using some psychoactive substances helped them improve performance at work activities. Here is what one of the respondents said:

***“****Khat also helps me to stay at work. For example, if I don’t chew while I wash clothes, I feel pain on my hand, and I become less committed to work.” [12*^*th*^*participant]*

Respondents used some psychoactive substances in order to avoid feeling of hunger or the need to eat.

*“Benefits I get from the substances? First, it suppresses hunger; especially, ‘amag’ and khat serve this purpose.” [6*^*th*^*participant]*

*“When using khat I don’t feel any cold or any hunger; I can stay days without having the need to eat.” [9*^*th*^*participant]*

Some respondents used the substances for the purpose of having the experience of pleasure.

*“Using those substances gives me pleasure.” [1*^*st*^*participant]*

*“Cannabis gives love and peace to me; I will be in the best mood when I use it.” [6*^*th*^*participant]*

Protection from cold weather was one of the benefits they got from using substances, as well as the reason to use substances on continuous basis.

*“Amag” protects from chilly weather while being on the streets; the need for ‘amag’ and the amount consumed increases significantly during the rainy season.” [5*^*th*^*participant]*

*“Substances protect us from the cold weather on the streets; ‘amag’ is the best in this regard, and mandatory in that kind of weather.” [6*^*th*^*participant]*

Respondents also reported substances alleviated their pain.

*“One feels no pain from beatings by the police while he/she is in the effect of the “amag”. We feel the pain at the site of the beaten body part later on when the effect of the “amag” wanes and clears.” [5*^*th*^*participant]*

Respondents used substances to stay awake during the night for some reason.

*“It helps to keep me awake during the night when there is the need to be vigilant against any danger which could happen to me … Some of the street people do ‘mela’, that means they steal property, during the night; they use psychoactive substances to stay awake for this purpose. Others use it to stay awake during the night to beg money from people who pass by … . There are thieves who steal little children; so to prevent my baby from being stolen I use substances like khat.” [8*^*th*^*participant]*

*“I use khat to stay awake all night for purpose of moving around the city and beg; the khat keeps us awake all night. We chew khat from 7 PM till 5 AM; we call this process “qatira” … Khat and cigarettes become very useful when conducting the ‘qatira.” [5*^*th*^*participant]*

*“Khat and ‘amag’ also help us stay awake during the night when it is necessary. For example, sometimes there are sick people whom we should attend during the night; during that time we consume khat and ‘amag.” [6*^*th*^*participant]*

### Harms of substances

The other category identified from the data analysis was the harms participants experienced from the use of substances.

Untimely death was mentioned by participants to be the harm they know about using substances. One of the participants said the following:

*“I hated street life because I witnessed the deaths of several of my friends. I remember of a friend who died from the overdose and adverse effect of inhaling the substance ‘amag.’ I also have seen little children die from the adverse effects of ‘amag.” [6*^*th*^*participant]*

Respondents experienced health problems due to the use of substances in their life as homeless.

*“ … …… … . I drank more than 20 glasses (around 330 ml each) after which I became ill and was taken to hospital unconscious.” [1*^*st*^*participant]*

*“ ......... I was usually worried about my use of “amag” because it is the most dangerous of all. It damages the lung; I saw users spit blood after inhaling “amag.” [5*^*th*^*participant]*

Dependence was another harm they experienced from using substances.

*“I usually cannot open my eye early in the morning unless I smoke cigarette; once I smoke, my eye opens up to my surprise.” [5*^*th*^*participant]*

### Means of obtaining substances

The other major category which resulted from data analysis was the means of obtaining a supply of the psychoactive substances which the participants had.

Respondents allocated all the available money to buying substances than to any other purpose. They said the following:

*“Whenever I get money, instead of food, it is on khat that I want to spend it.” [9*^*th*^*participant]*

*“Now, if I have limited amount of money, I prefer using it to buy those substances than buy food with it.” [8*^*th*^*participant]*

At times, the participants had to borrow money to buy substances.

*“I will borrow money to buy khat if I have no money.” [1*^*st*^*participant]*

Participants reported they more often had to collect whatever is left off the substances after use by people.

*“ … … We move around parts of the city where there are bars and nightclubs and wait for the remains of alcohol in bottles and glasses left by the customers. When the employees of the bars collect the bottles and glasses with the remaining alcohol in it out we empty the contents in to our plastic containers (we call alcohol we collect this way “chile”).” [5*^*th*^*participant]*

*“We collect khat left after people consumed the tip of it and they have thrown the rest (the ‘geraba’).” [8*^*th*^*participant]*

Some respondents were involved in the retail of substances themselves (or, they became dealers of the substance), and that helped them secure their supply of the substances.

*“ … ……… … ... I also was engaged in the trade of ‘amag’; I buy the merchandise from the shops and retail it to those who want it on the streets. I get a substantial amount of money from such trade, besides securing my own supply.” [6*^*th*^*participant]*

Some respondents worked in prostitution to get money for their expenses. They used the money to buy psychoactive substances.

*“When I was 19 years old I started to do prostitution; prostitution was the last thing I wanted to do, but helped me earn money for my expenses.” [1*^*st*^*participant]*

## Discussion

Our study is unique from similar studies conducted in various places previously. It is different in that it is more comprehensive and explored in-depth several aspects of substance use among homeless women. Our study explored factors for onset of substance use, perceived benefits and reasons for continued use of the substances, the harms from the use of substances, and the means of obtaining supply of the substances. The study also is unique in that it focused on substance use among homeless women, a part of the homeless population which has not been given adequate attention by the research community. We identified no previous studies of substance use conducted among homeless women to compare our study with; therefore, we will compare our findings with previous studies conducted among population of homeless people in general.

Most of the reasons for beginning use of psychoactive substances identified in the current study are similar to findings of previous studies. Our study found out that “identification with street culture” was one reason for beginning use of substances among the female homeless people. This finding is similar to the finding of a previous study [51]. The ‘street culture’ includes ways of life such as engaging in accepted practices for earning money (such as panhandling), adopting unique slang language, and developing strategies to prevent victimization [51]. Using, even abusing substances is often viewed as a “normal” practice by those identifying with street culture. Our study identified additional reasons why homeless women begin using substances after they become homeless. Peer pressure from other members of the homeless community was an important reason. Occurrence of overwhelming stressful life event was also the other reason for beginning to use substances after women become homeless. Some were born as homeless, and were raised on the streets; such women did not remember how they first used the substances but reported they were users of the substances ever since they remembered.

Our study also identified that homeless women frequently used substances in order to cope with stress; this finding is in agreement with a study conducted previously among young homeless people [52]. It should be noted, however, that female homeless live under increased and higher stressful situation than homeless males [53]; in fact, the current study has identified extreme levels of stressful life events occurring to homeless women to result in the use of substances as a means of coping. Substances were used by homeless women in order to numb unfavorable feelings which result from the negative effects of traumatic experiences; this finding from the current study is similar to that of a previous study [17]. In our study, homeless women used substances to avoid feelings of cold and to suppress feeling of hunger; this finding is supported by findings of a previous study [54]. Previous studies showed that some drugs were used by the street homeless to help them stay awake for extended periods, especially at night when the chances of victimization increased (55, 56). This finding is supported by the finding of the current study. In addition to safety concerns, homeless women in our study wanted to stay awake during the night for the purpose of income generating activities, and to take care of their sick or vulnerable associates. Drugs also provided the homeless a means of escape from the physical and emotional pain associated with surviving on the street. This is a finding identified in our study, as well as in a previous study (57). However, the current study has identified additional reasons for the habitual use of substances. Those additional reasons were presence of dependence on the substances, obtaining a pleasurable experience from the substances, and and using substances to increase performance at work activities.

Generally, in conformation with previous studies, our study shows that drug use is, for most part, an adaptive response to unpleasant and stressful environment. For that reason, homeless women pay all they have in order to obtain the substances; our study participants allocated all the available money for buying substances, and they even gave substances preference to food. The current study has identified additional means of obtaining supply of substances available for the homeless women. They borrowed money from others, they worked as prostitutes to make money and afford to buy substances, as well as move around the city streets searching for leftover of the substances on streets and in bars. Homeless women also get involved in retail trade and as dealers of the substances to be able to secure their own supplies of the substances. However, like previous studies [32], our study showed that substance use created newer problems for the homeless women. In addition to the hardships of obtaining supplies of the substances, their use created additional harms. Our study showed that untimely death was a possibility due to substance use, a finding supported by a previous study [41]. Our findings support findings by previous studies showing that drug users suffered from numerous adverse health effects, including overdoses, psychiatric conditions, and infectious diseases [39, 40, 42]. Our study identified health problems and harm from overdose were experienced by homeless women who use psychoactive substances. Becoming dependent on the substances was an additional finding from the current study, which was identified as harm which results from use of psychoactive substances on the streets.

### Limitations

We acknowledge that the previous knowledge and understanding of the investigators about the topic could have influenced the findings and conclusions of the study. While the sample size might be considered low, it was decided by the theoretical saturation of the information from respondents and believed to be adequate for the purpose of the study. The study is an exploratory qualitative study, and as such the results should be interpreted considering the limitation that the study participants are not representative of all homeless women in Addis Ababa.

## Conclusion

Our study participants started consuming substances after they became homeless due to various reasons. The reasons include the need to cope with stress and adversity, to conform to street culture, due to peer pressure, etc. Once they began using substances, they used them on continued basis in order to cope with challenges of homeless life, to numb unfavorable physical and emotional feelings, to obtain feeling of pleasure, to stay awake and vigilant of possible danger and for income generating activities, because of addiction, and to get protection from the feelings of cold and hunger. The study participants suffered health problems, addiction and untimely death due to their use of psychoactive substances. Despite the perceived harms substances caused to them, the participants applied all the means they had to obtain the substances. There was the preference of allocating available money for buying substances, begging, prostitution, debt, as well as getting involved in dealing with the substances. Further studies are recommended to confirm findings from this study, including the conduction of quantitative study using a representative sample.

## Data Availability

The datasets generated during and/or analyzed during the current study are not publicly available due to ethical restrictions and for protection of participant privacy but are available from the corresponding author on reasonable request.
